# Complete Genome Sequence of *Corynebacterium urealyticum* Strain DSM 7111, Isolated from a 9-Year-Old Patient with Alkaline-Encrusted Cystitis

**DOI:** 10.1128/genomeA.00264-13

**Published:** 2013-05-23

**Authors:** Luis C. Guimarães, Siomar C. Soares, Andreas Albersmeier, Jochen Blom, Sebastian Jaenicke, Vasco Azevedo, Francisco Soriano, Andreas Tauch, Eva Trost

**Affiliations:** Institut für Genomforschung und Systembiologie, Centrum für Biotechnologie, Universität Bielefeld, Bielefeld, Germanya; CLIB Graduate Cluster Industrial Biotechnology, Centrum für Biotechnologie, Universität Bielefeld, Bielefeld, Germanyb; Laboratório de Genética Celular e Molecular, Departamento de Biologia Geral, Instituto de Ciências Biológicas, Universidade Federal de Minas Gerais, Belo Horizonte, MG, Brazilc; Public Health School of Physiotherapy, Autonomous University of Madrid, Madrid, Spaind

## Abstract

*Corynebacterium urealyticum* is a common skin colonizer with potent urease activity. It is clinically recognized as an opportunistic pathogen causing urinary tract infections. The annotated genome sequence of strain DSM 7111, isolated from the urine of a young boy with an ectopic kidney, provides new insights into the pathomechanisms of this bacterium.

## GENOME ANNOUNCEMENT

*Corynebacterium urealyticum* (formerly *Corynebacterium* group D2) is commonly isolated from the skin of hospitalized patients who are receiving broad-spectrum antibiotics (1). This bacterium has been well known for more than 30 years as an opportunistic pathogen causing mainly acute or encrusted cystitis, encrusted pyelitis, and pyelonephritis (1). It has also been detected as an uncommon pathogen in the urinary tract of small animals, such as cats and dogs (2, 3). The urease activity of *C. urealyticum* plays a key role in the pathogenesis of urinary tract infections and in the formation of struvite (magnesium ammonium phosphate) stones (4). In cases of chronic infections the medical treatment requires the administration of drugs and additional surgical intervention (1). The efficiency and outcome of the treatment are often affected by multiple resistances of *C. urealyticum* to a broad range of antibiotics (5). Antibiotic resistance genes detected in the genome of the clinical isolate *C. urealyticum* strain DSM 7109 are part of mobile DNA elements, which suggests that horizontal gene transfer is the main mechanism contributing to the development of multidrug resistance in this species (6).

*C*. *urealyticum* DSM 7111 (ATCC 43040) was isolated from urine samples of a 9-year-old patient with an ectopic kidney (7). Genomic DNA was purified from an overnight culture as described previously (6). Genome sequencing was performed in a combined approach using the 454 Genome Sequencer FLX system (Roche Applied Science) and the Ion Torrent PGM Sequencer (Life Technologies). Pyrosequencing resulted in 799,528 reads and 80,374,097 detected bases, whereas semiconductor sequencing revealed 484,970 reads and 56,643,033 bases. The reads were assembled with the GS *de novo* Assembler (version 2.6) and the G4ALL software package (version 1.0.5) (http://g4all.sourceforge.net/) to yield 74 contigs sequenced with 59-fold coverage. The remaining gaps in the genome sequence were closed by PCR amplification and subsequent sequencing of the DNA fragments with an ABI 3730xl DNA Analyzer (Life Technologies). The deduced chromosome of *C. urealyticum* DSM 7111 has a size of 2,316,065 bp with an average G+C content of 64.24%. The manual annotation of the genome sequence was supported by the GenDB platform (8) and the REGANOR gene prediction server (9). Putative pseudogenes were identified with the CLC Genomics Workbench (CLC bio). Subsequent analysis of the sequence data followed established bioinformatic protocols for corynebacterial genomes (6). The annotation of the *C. urealyticum* DSM 7111 genome sequence revealed 1,935 protein-coding genes, 3 rRNA operons, and 54 tRNAs. The EDGAR software (10) detected 79 genes that are specific for *C. urealyticum* DSM 7111 and not present in the genome of the type strain *C. urealyticum* DSM 7109. Some of these strain-specific genes are clustered and encode a siderophore biosynthesis pathway, an iron ABC transport system, and a multicopper oxidase system, suggesting a different iron acquisition mechanism in *C. urealyticum* DSM 7111. Moreover, a putative phenylacetic acid degradation pathway might contribute to the virulence of *C. urealyticum* DSM 7111.

### Nucleotide sequence accession number.

The genome project has been deposited in GenBank under the accession number CP004085.
